# Antibacterial and resistance-modifying activities of thymoquinone against oral pathogens

**DOI:** 10.1186/1476-0711-10-29

**Published:** 2011-06-27

**Authors:** Bochra Kouidhi, Tarek Zmantar, Hanene Jrah, Yosra Souiden, Kamel Chaieb, Kacem Mahdouani, Amina Bakhrouf

**Affiliations:** 1 Laboratory of Analyses, Treatments and Valorisation of Environmental Wastes and Products, Faculty of Pharmacy, Monastir University, Avicenne Street 5000 Monastir, Tunisia; 2 Laboratory of Molecular Biology, Kairouan Hospital, Tunisia; 3 Research Unit of Biology and Genetics of Hematological and Auto-immune Diseases, Faculty of Pharmacy, Monastir University, Avicenne Street 5000 Monastir, Tunisia

## Abstract

**Background:**

The presence of resistant bacteria in the oral cavity can be the major cause of dental antibiotic prophylaxis failure. Multidrug efflux has been described for many organisms, including bacteria and fungi as part of their drugs resistance strategy. The discovery of a new efflux pump inhibitor could extend the useful lifetime of some antibiotics.

**Methods:**

In this study, the MICs of thymoquinone (TQ), tetracycline and benzalkonium chloride (BC) were determined in absence and in presence of a sub-MIC doses of thymoquinone (1/2 MIC). In addition the 4,6-diamidino-2-phenylindole (DAPI) efflux assay was carried out to determine the effect of TQ on DAPI cells accumulation.

**Results:**

TQ induced a selective antimicrobial activity. Its synergic effect resulted in at least a 4-fold potentiation of the tested antibiotics and antiseptic. In addition, TQ inhibited the DAPI efflux activity in a concentration-dependent manner. The rate of DAPI accumulation in clinical isolates was enhanced with TQ (0 to 200 μg/ml). There is also a decrease in loss of DAPI from bacteria in the presence of TQ. The concentration causing 50% of DAPI efflux inhibition after 15 minutes was approximately 59 μg/ml for *Pseudomonas aeroginosa *and 100 μg/ml and *Staphylococcus aureus *respectively.

**Conclusions:**

TQ possesses a selective antibacterial activity against oral bacteria. It is therefore suggested that TQ could be used as a source of natural products with resistance-modifying activity. Further investigation is needed to assess their clinical relevance.

## Background

The human oral cavity is an habitat for about 500 cultivable and non-cultivable bacterial species [1]. They have also been implicated in the aetiology of a number of systemic diseases such as infective endocarditis, respiratory infections and cardiovascular diseases [2-4]. *Streptococcus *spp. have been implicated as primary causative agents of dental caries, especially, *Streptococcus mutans *and *Streptococcus sobrinus *[5,6].

Bacteria are exceptionally adept at acquiring resistance to antibiotics and antiseptic agents [7]. Sweeney et al., [8] reported the resistance of oral bacteria to pencillins, tetracycline and macrolides. The difficulty in treating multi-resistance bacterial infections is compounded by the fact that many strains also possess efflux pumps (e.g. TetK and MsrA, NorA and QacA) which confer resistance to various antibiotics and antiseptics [9].

Natural compounds have been recently investigated as promising agents for the prevention of dental caries [10]. *Nigella sativa *L. is an annual herbaceous plant belonging to the Ranunculaceae family growing in countries bordering the Mediterranean Sea [11]. The seeds of *Nigella sativa *L. have been particularly used in the traditional Arab herbal medicine for the treatment of various diseases [12]. Many biological activities of *N. sativa *seeds have been reported, including: antibacterial, antifungal, anti-tumor, and hypotensive [13-16].

Thymoquinone was the bioactive constituent of the volatile oil of *N. sativa *[17]. Moreover, it has been reported that TQ have antibacterial potency and its activity can enhance antibiotic actions especially against *S. aureus *[18].

The rapid spread of bacteria expressing multidrug resistance has necessitated the discovery of new antibacterial and resistance-modifying agents. Efflux pumps have been known to extrude structurally diverse compounds, including antibiotics and antiseptics used in a clinical setting [9]. The combination of a broad-spectrum multidrug-resistant (MDR) pump inhibitor with antibiotics could reduce the morbidity and mortality that might result from a delay in the institution of effective therapy for serious *S. aureus *infections [19]. Antimicrobial and efflux pumps inhibiting activities of natural compounds have been reported for several natural based products such as rosemary, kaempferol, propolis and aqueous khat extracts [20-23].

The aim of this study was to evaluate the *in vitro *inhibitory and resistance-modifying properties of TQ alone or in combination with tetracycline and BC against a panel of pathogenic bacteria.

## Methods

### Microorganisms

The cariogenic strains (n = 16) used in this study were isolated from Tunisian children suffering for dental caries (Monastir, Center of Tunisia). The strains were isolated on blood agar plates supplemented with 5% sheep blood and identified by conventional methods.

11 reference strains were further included in this study. All the used bacteria were listed in Table 1.

**Table 1:** Minimum inhibitory and minimum bactericidal concentrations in μg/ml of thymoquinone and tetracycline and their combination

	MIC TQ	MBC TQ	MIC TET	MBC TET	^a^MIC TET + 1/2MIC TQ	MBC TET + 1/2MIC TQ
**References strains**						
*Bacillus cereus *ATCC 14579	8	8	2	8	0.5 (4)	4 (2)
*Escherichia coli *ATCC 35218	512	512	4	16	2 (2)	64 (4)
*Enterococcus faecalis *ATCC 29212	32	128	256	512	128 (2)	64 (8)
*Salmonella enterica *serovar Typhimurium ATCC 1408	256	512	128	256	32 (4)	128 (2)
*Staphylococcus aureus *ATCC 25923	8	16	4	8	0.5 (8)	4 (2)
*Staphylococcus epidermidis *CIP 106510	8	8	8	16	4 (2)	64 (4)
*Listeria monocytogenes *ATCC 19115	32	128	1	4	1 (NC)	4 (NC)
*Micrococcus luteus *NCIMB 8166	8	64	32	64	8 (4)	16 (4)
*Pseudomonas aeruginosa *ATCC 27853	>512	>512	64	128	32 (2)	256 (2)
*Vibrio alginolyticus *ATCC 33787	512	512	256	512	128 (2)	256 (2)
*Vibrio paraheamolyticus *ATCC 17802	32	64	4	32	0.5 (8)	8 (4)
**Oral strains**						
*E. faecalis *B281	256	256	64	256	64 (NC)	128 (2)
*Gemella haemolysans *B234	128	128	512	256	256 (2)	256 (NC)
*Staphylococcus aureus *B73	256	256	64	128	16 (4)	16 (8)
*Staphylococcus aureus *B285	8	32	4	4	4 (NC)	8 (2)
*Staphylococcus aureus *B291	256	256	128	128	32 (4)	64 (2)
*Staphylococcus aureus *B289	16	16	4	4	<0.5 (>8)	2 (2)
*Staphylococcus aureus *B456	16	32	32	128	4 (8)	16 (8)
*Staphylococcus aureus *B244	16	32	32	32	4 (8)	8 (4)
*Staphylococcus aureus *B364	256	256	4	8	8 (2)	8 (NC)
*Staphylococcus aureus *B398	8	16	16	16	4 (4)	4 (4)
*Streptococcus anginosus *B486	64	64	128	256	64 (2)	128 (2)
*Streptococcus constellatus *B629	32	64	<0,5	4	<0.5 (NC)	4 (NC)
*Streptococcus mitis *B627	128	256	128	256	32 (4)	128 (2)
*Streptococcus mutans *B509	16	16	<0,5	8	<0.5 (NC)	4 (2)
*Streptococcus oralis *B634	32	64	128	128	32 (4)	64 (2)
*Streptococcus salivarius *B468	16	32	4	16	2 (2)	8 (2)

^a^Fold reductions are given in parentheses; NC, no change; TQ, thymoquinone; TET, Tetracycline

### Chemicals used

All the media used in this study were purchased from Biorad (France), thymoquinone from Sigma-Aldrich (Switzerland) and benzalkonium from Acros organics (USA).

### Minimum inhibitory concentration (MIC) value determination assay

The broth microdilution method was used to determine the minimum inhibitory concentration (MIC) of TQ against the tested strains as recommended by the Clinical and Laboratory Standards Institute (CLSI) [24]. Cells (10^6^/ml) were inoculated into Mueller-Hinton broth and dispensed at 0.2 ml/well in 96-well microtiter plates. The TQ was properly prepared and transferred to each microplate well in order to obtain a twofold serial dilution ranging from 0.5 to 256 μg/ml. The inocula (10 μL) containing 5 10^5 ^CFU of each microorganism were added to each well. A number of wells was reserved in each plate for sterility control (no inoculate added) and inocula viability (no TQ added). All MICs tests were repeated three folds in separate times. Plates were incubated at 37°C for 24 h and bacterial growth was evaluated by the presence of turbidity and a pellet on the well bottom. MIC value was defined as the lowest concentration of the antimicrobial compound that had no macroscopically visible growth.

### Resistance modifying assay

To test the resistance-modifying activity of TQ, the tetracycline and benzalkonium chloride (BC) MICs ranging from 0.5 to 256 μg/ml were determined against the selected strains with or without TQ at 1/2 of its MIC value using the microtiter plates assay [23]. All experiments were carried out three times.

### Minimum bactericidal concentration (MBC) value determination assay

To determine the MBCs values, 10 μl from each well of broth with no visible growth were removed and inoculated on Muller Hinton agar plates. After 18-24 h of incubation at 37°C, the number of surviving bacteria was noted. MBC value was defined as the lowest concentration of compounds (TQ, tetracycline and BC) needed to kill 99% of bacteria. Each experiment was repeated at least twice [25].

### Efflux assay

The 4,6-diamidino-2-phenylindole (DAPI) efflux assay was carried out as described previously [26]. Briefly, cells were grown in 20 ml of Luria-Bertani (LB) broth until the optical density at 650 nm reached 0.7 units. The cells were washed with modified Tanaka buffer and were resuspended in the same buffer containing 5 μM of DAPI and 1 mM 2,4-dinitrophenol (DNP), and incubated at 37°C for 10 h [27,28]. DNP, which is a well-known conductor of protons across the cytoplasmic membrane, was used to de-energize the cells [29].

Similar steps were repeated to obtain an optical density of 0.4 units at 650 nm. The fluorescence of DAPI was measured at excitation and emission wavelengths of 355 and 457 nm respectively, with a Spectrofluorophotometer, model RF-5301PC (Shimadzu). The fluorescence intensity of DAPI is higher when DAPI binds to DNA molecules. Thus, the efflux of DAPI from the cell can be monitored by the detection of a decrease in the level of fluorescence over time. After incubation of the cell suspension at 37°C for 5 min, glucose (20 mM) was added as an energy source to monitor the efflux of DAPI.

To evaluate the effects of TQ on the efflux of DAPI, cell suspensions were prepared in the same way as described above. Cell suspensions were pre-incubated for 5 min at 37°C with different concentrations of TQ (0 to 200 μg/ml) prior to the addition of glucose.

## Results

### Antibacterial activity of thymoquinone

The antibacterial activities of TQ against the tested strains were shown in Table 1. TQ demonstrated a selective antimicrobial property. Seven out of 16 oral strains, particularly *Staphylococcus aureus *(B285, B289, B456, B244 and B398), *Streptococcus mutans *(B509), *Streptococcus salivarius *(B468), and four out of 11 laboratory reference strains, which consist of *Staphylococcus epidermidis *CIP 106510, *Staphylococcus aureus *ATCC 25923 *Micrococcus luteus *NCIMB 8166 and *Bacillus cereus *ATCC 14579 were sensitive to TQ with MIC and MBC values ranging from 8 to 64 μg/ml. Six clinical and four reference strains were resistant to TQ with MIC values ranged between 128 to 512 μg/ml (Table 1 and 2). Furthermore, the most resistant strain was *Pseudomonas aeroginosa *with a MIC of value >512 μg/ml.

**Table 2:** Minimum inhibitory and minimum bactericidal concentrations in μg/ml of benzalkonium with and without thymoquinone supplementation.

Strains	MIC BC	MBC BC	^a^MIC BC + 1/2MIC TQ	^a^MBC BC +1/2 MIC TQ
**References strains**				
*B. cereus *ATCC 14579	16	32	4 (4)	8 (4)
*E. coli *ATCC 35218	16	32	16 (NC)	16 (2)
*E. faecalis *ATCC 29212	8	16	4 (2)	4 (4)
*L. monocytogenes *ATCC 19115	1	4	1 (NC)	2 (2)
*M. luteus *NCIMB 8166	16	16	2 (8)	4 (4)
*S. enterica *serovar Typhimurium ATCC 1408	32	64	32 (NC)	64 (NC)
*S. aureus *ATCC 25923	16	32	2 (8)	4 (8)
*S. epidermidis *CIP 106510	4	8	2 (2)	4 (2)
*P. aeruginosa *ATCC 27853	128	256	128 (NC)	256 (NC)
*V. alginolyticus *ATCC 33787	32	64	16 (2)	32 (2)
*V. paraheamolyticus *ATCC 17802	32	128	4 (8)	16 (8)
**Oral strains**				
*E. faecalis *B281	256	>256	256 (NC)	>256 (NC)
*Gemella haemolysans *B234	8	32	4 (2)	8 (4)
*S. aureus *B73	16	32	2 (8)	8 (4)
*S. aureus *B285	8	16	2 (4)	4 (4)
*S. aureus *B291	16	16	2 (2)	4 (4)
*S. aureus *B289	8	16	<1 (>8)	2 (8)
*S. aureus *B456	4	8	<1 (>8)	2 (4)
*S. aureus *B244	2	8	<1 (>8)	2 (4)
*S. aureus *B364	64	128	8 (>8)	32 (4)
*S. aureus *B398	4	8	<1 (>8)	2 (4)
*S. anginosus *B486	8	16	4 (2)	8 (2)
*S. constellatus *B629	4	16	2 (2)	4 (4)
*S. mitis *B627	4	8	4 (NC)	16 (2)
*S. mutans *B509	4	8	2 (2)	4 (2)
*S. oralis *B634	>256	>256	>256 (NC)	>256 (NC)
*S. salivarius *B468	8	16	4 (2)	8 (2)

^a^Fold reductions are given in parentheses; NC, no change; TQ, Thymoquinone; BC, benzalkonium chloride

### Resistance modifying properties of thymoquinone

Data presented in Table 1 showed that the supplementation of TQ (at 1/2 MIC) induces the highest decrease of eight-fold MIC value of tetracycline against *S. aureus *ATCC 25923, *S. aureus *B289 and *Vibrio paraheamolyticus *ATCC 17802. Additionally, a four-fold reduction of tetracycline MIC value was observed against five clinical and three reference strains. A two-fold potentiation of the tetracycline activity against five reference and four clinical isolates was also noted (Table 1).

Similarly, an eight-fold potentiation of BC with the addition of TQ (at 1/2 MIC) was recorded against the same three reference strains with additional four *S. aureus *isolated from the oral cavity. Furthermore, a four-fold BC MIC reduction was noted against *B. cereus *ATCC 14579 and *S. aureus *B285 (Table 2).

### Efflux-mediated properties of thymoquinone

The efficiency of efflux pumps for which DAPI is a substrate has been assessed fluorometrically. In this study, we investigated the effect of TQ on the DAPI efflux activity. The fluorescence of DAPI increases when it binds to DNA. Glucose was added as an energy source to the assay mixture, which monitor the DAPI efflux.

**Figure 1:**
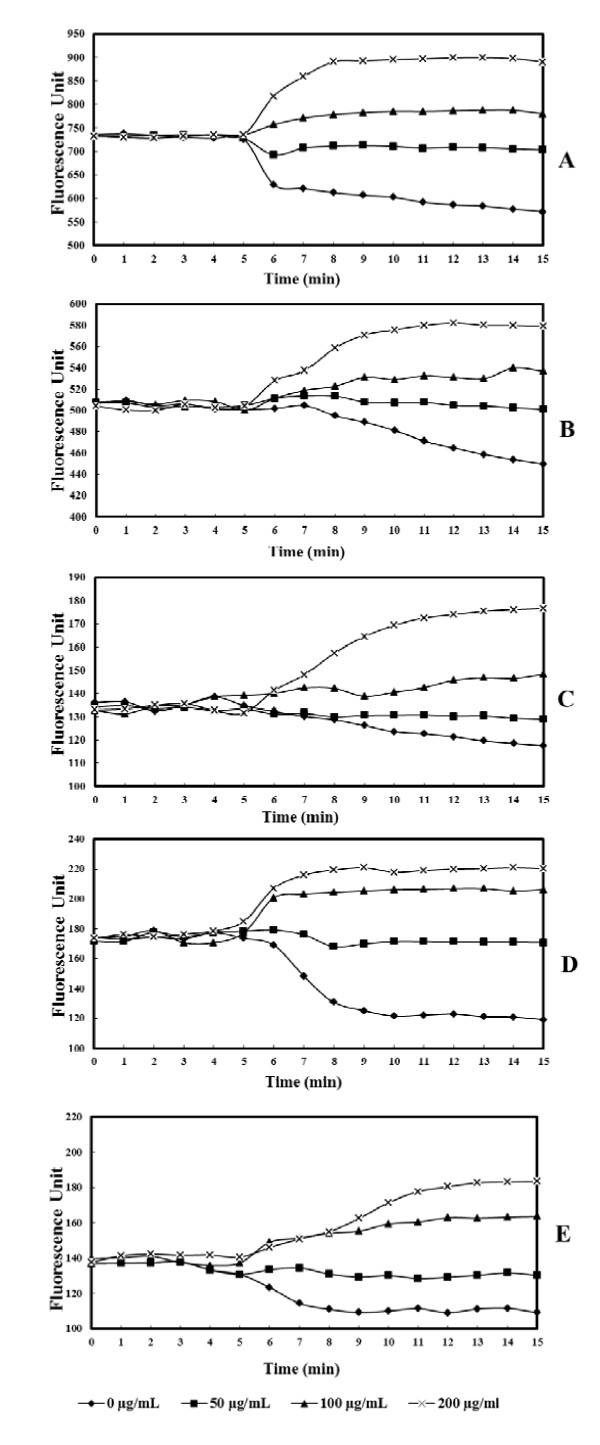
**The levels of accumulation of 4,6-diamidino-2-phenylindole in pathogenic bacteria alone or in the presence of various concentration of thymoquinone (0, 50, 100 and 200 μg/mL)**: A, *Bacillus cereus *ATCC 14579; B, *Enterococcus faecalis *ATCC 29212; C, *Vibrio parahemolyticus *ATCC 17802; D, *Pseudomonas aeruginosa *ATCC 27853; E, *Staphylococcus aureus *ATCC 25923.

In the absence of thymoquinone, the addition of glucose after 5 min of experience induced a significant decrease of fluorescence represented by an x symbol in Figure 1(A, B, C, D) and 1E. However, TQ supplementation inhibit the DAPI efflux

**Figure 2:**
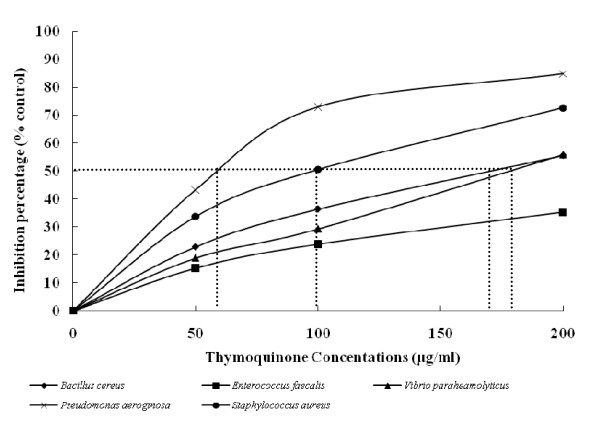
**Inhibition of DAPI efflux activity by thymoquinone by different bacteria**. Various concentrations of thymoquinone were added, and the mixture was preincubated with the cells for 5 min. Glucose (final concentration, 20 mM) was added to initiate the assay. The relative initial velocity of DAPI efflux was measured. The initial velocity observed in the absence of Thymoquinone was set at 100%. Dotted lines indicate IC_50_.

As shown in Figure 2, the intracellular accumulation of DAPI was influenced by TQ in a concentration-dependent manner. Low doses of TQ reduced the DAPI efflux whereas higher doses showed a total inhibition of efflux and even a DAPI accumulation reflected in an increase of fluorescence. As the concentration of TQ increased, the fluorescence was increased indicating the concentration-dependant inhibition of DAPI efflux through active pumps.

The concentration causing 50% of efflux inhibition after 15 minutes was approximately 59 μg/ml, 100 μg/ml, 169 μg/ml and 177 μg/ml 177 μg/ml against *P. aeroginosa*, *S. aureus*, *B. cereus *and *V. parahaemolyticus*, respectively (Figure 2).

A similar efflux inhibition was observed for *Enterococcus faecalis*. However, the maximum of inhibition observed was lower than 30%. These data indicate that at low concentrations, TQ is very effective as an inhibitor of DAPI efflux in the tested bacteria (Figure 2).

## Discussion

One of the most important antibiotic resistance mechanisms is the expression of efflux pumps. The search of a new efflux pump inhibitors (EPIs) is necessary to combat the emergence of MDR strains [30].

Data presented in Table 1 revealed a selective antibacterial property of TQ. Seven reference strains and 10 oral isolates were sensitive to TQ with MIC values ranged from 8 to 64 μg/ml while the remaining four Gram-negative reference strains and six oral bacteria were resistant against TQ with MIC values ranging from 128 to 512 μg/ml. These results support a previous study which reported an effective and inactive potency of TQ against Gram-positive and Gram-negative bacteria, respectively [18].

Efflux is an important mechanism of resistance in many clinically relevant pathogens, notably, *Streptococcus pneumoniae *and *Pseudomonas aeruginosa *[9,31]. The efflux pumps (EPs) are proteins of bacterial membranes which extrude antibiotics and other antimicrobial agents from the cell [32]. These EPs can transport drugs through the bacterial envelope and limit the intracellular accumulation of toxic compounds, such as antibiotics, antimicrobial peptides, metals and detergents [32]. It has been reported that plants provide a rich source of efflux pumps inhibitors (EPIs) [19,30]. Therefore, there is an urgent need for novel drugs with new modes of action, such as EPIs, to prevent the rise of MDR bacteria [20]. EPI activities of natural compounds have been reported elsewhere [20,21,23].

Data presented in Table 1 and 2 showed the potential of TQ to reduce at least a 4-fold the tetracycline and BC MICs value. Similar effect of TQ with other antibiotics has been previously reported [18].

In the case of BC an 8-fold reduction in MICs values were observed particularly for *Staphylococcus aureus *and *Vibrio paraheamolyticus *(Table 2).

The modulating activity of TQ was referred as "Efflux Pump Inhibitors". This expression was adopted for compounds isolated from *Lycopus europaeus *and *Rosmarinus officinalis *which modulate resistance of *S. aureus *to tetracycline and erythromycin [19,20]. To the best of our knowledge, this is the first report on resistance modifying activity of TQ against resistant oral bacteria.

In a DAPI accumulation assay, we compared the levels of DAPI accumulation in five pathogenic bacteria treated by TQ (0 to 200 μg/ml) during 15 minutes (Figure 1A, B, C, D and 1E). Our data revealed that the addition of TQ induced the increased of DAPI accumulation in the treated strains. The inhibition of DAPI efflux via a number of pumps transporters has been already reported for *E. faecalis *[26].

We noted also that accumulation of DAPI was further increased following TQ supplementation. As found previously, the MICs of the antibiotics and BC were also much lower following TQ supplementation. A relative difference in increased accumulation of DAPI in the presence of various TQ concentrations was noted (Figure 2).

These two observations presume the modulating activity of TQ through pumps efflux inhibition leading to antibiotic accumulation in the cells enhancing their effects at lower doses. Furthermore DAPI is known to be substrates for many efflux pumps and no system other than multi-drugs efflux pumps are known to cause resistance to these agents [33-35]. So the inhibition of DAPI efflux supports the hypothesis of antibiotics modulating activity of thymoquinone through pump efflux inhibition.

Modulators of drug resistance would clearly have the benefit for the treatment of multidrug resistant strains for which the majority of therapeutic antibiotics have no further clinical use. Inhibitors of drug efflux mechanisms could, in combination, greatly extend the useful lifetime of older conventional antibiotics such as the tetracycline.

## Conclusion

We have demonstrated that TQ have antibacterial and resistance modifying activity. Thus, the results shown in the present report are encouraging although clinical controlled studies are needed to define the efficacy of TQ. These studies could determine the potential medical use of TQ in combination with selected antimicrobial drugs against bacterial infection.

Since bacteria may be resistant to several antimicrobial drugs, the synergism reported here is of relevance and TQ may constitute an alternative for treating infections related to these pathogens.

## Competing interests

The authors declare that they have no competing interests.

## Authors' contributions

BK is the primary author of the manuscript, planed the work, assisted in minimum inhibition concentration determination of TQ, tetracycline and BC together and conceived the DAPI efflux assay. TZ contributed in minimum inhibition concentration determination and helped in the writing of the manuscript. HJ participated in data acquisition and contributed in writing of the manuscript. YS helped in DAPI efflux determination, and participated in the writing of the manuscript. KM participated in data acquisition and helped to finalize the manuscript. AB provided funding, supervised the study, and helped to finalize the manuscript.

All the authors read and approved the final version of the manuscript.

## Financial competing interests

Higher education and scientific research in Tunisia through the laboratory of analyses, treatments and valorisation of environmental wastes and products, faculty of pharmacy, Monastir University, street Avicenne 5000 Monastir (Tunisia).
